# *Clostridium perfringens* Epsilon-Toxin Impairs the Barrier Function in MDCK Cell Monolayers in a Ca^2+^-Dependent Manner

**DOI:** 10.3390/toxins12050286

**Published:** 2020-04-30

**Authors:** Masahiro Nagahama, Soshi Seike, Sadayuki Ochi, Keiko Kobayashi, Masaya Takehara

**Affiliations:** 1 Department of Microbiology, Faculty of Pharmaceutical Sciences, Tokushima Bunri University, Yamashiro-cho, Tokushima 770-8514, Japan; kobakei@ph.bunri-u.ac.jp (K.K.); mtakehara@ph.bunri-u.ac.jp (M.T.); 2 Laboratory of Molecular Microbiological Science, Faculty of Pharmaceutical Sciences, Hiroshima International University, Kure, Hiroshima 737-0112, Japan; s-seike@hirokoku-u.ac.jp; 3 Faculty of Pharmacy, Yokohama University of Pharmacy, 601 Matano-cho, Totsuka-ku, Yokohama-shi, Kanagawa 245-0066, Japan; sadayuki.ochi@hamayaku.ac.jp

**Keywords:** *C. perfringens* epsilon-toxin, barrier integrity, oligomer formation, cofilin, Ca^2+^ influx

## Abstract

Epsilon-toxin produced by *Clostridium perfringens* significantly contributes to the pathogeneses of enterotoxemia in ruminants and multiple sclerosis in humans. Epsilon-toxin forms a heptameric oligomer in the host cell membrane, promoting cell disruption. Here, we investigate the effect of epsilon-toxin on epithelial barrier functions. Epsilon-toxin impairs the barrier integrity of Madin-Darby Canine Kidney (MDCK) cells, as demonstrated by decreased transepithelial electrical resistance (TEER), increased paracellular flux marker permeability, and the decreased cellular localization of junctional proteins, such as occludin, ZO-1, and claudin-1. U73122, an endogenous phospholipase C (PLC) inhibitor, inhibited the decrease in TEER and the increase in the permeability of flux marker induced by epsilon-toxin. The application of epsilon-toxin to MDCK cells resulted in the biphasic formation of 1,2-diacylglycerol (DAG) and inositol-1,4,5-triphosphate (IP_3_). U73122 blocked the formation of DAG and IP_3_ induced by the toxin. Epsilon-toxin also specifically activated endogenous PLC-γ1. Epsilon-toxin dose-dependently increased the cytosolic calcium ion concentration ([Ca^2+^]i). The toxin-induced elevation of [Ca^2+^]i was inhibited by U73122. Cofilin is a key regulator of actin cytoskeleton turnover and tight-junction (TJ) permeability regulation. Epsilon-toxin caused cofilin dephosphorylation. These results demonstrate that epsilon-toxin induces Ca^2+^ influx through activating the phosphorylation of PLC-γ1 and then causes TJ opening accompanied by cofilin dephosphorylation.

## 1. Introduction

Epsilon-toxin, secreted by *Clostridium perfringens* types B and D, is a pore-forming toxin responsible for enteritis and enterotoxemia in sheep and other animals during *C. perfringens* infection [1,2,3,4]. The toxin also plays an important role in the pathogenesis of multiple sclerosis (MS) in humans [5,6,7]. 

Epsilon-toxin is secreted by intestinal tract bacteria as a relatively inactive prototoxin (32.9 kDa molecular weight). Prototoxin cleavage by proteolytic enzymes such as trypsin was shown to remove N- and C-termini peptides, leading to its activation (epsilon-toxin) [2,3,4]. The toxin exhibits lethal and dermonecrotic activities and induces increases in blood pressure [1,2]. Epsilon-toxin can also cause substantial damage to the intestinal epithelia, and is thought to penetrate the bloodstream to disperse throughout the body [8,9]. The toxin causes pathological damage, principally in the brains and kidneys of poisoned mice [10,11,12,13]. Epsilon-toxin is the third most potent clostridial toxin after botulinum and tetanus toxins [14], and is listed as a category B poisonous agent by the Centers for Disease Control [4]. 

Epsilon-toxin is a member of the aerolysin-like β-pore-forming toxin family [15]. The toxin forms oligomeric pores in lipid bilayers and in the plasma membranes of sensitive cells [16,17,18]. We demonstrated that the membrane fluidity in lipid bilayers is responsible for the pore formation by epsilon-toxin [17]. The cellular mode of the action of epsilon-toxin involves binding to specific receptors on the plasma membrane of sensitive cells, oligomer formation, and penetration into the plasma membrane. The toxin induces increased cell permeability and the reducion of cytosolic ATP and K^+^ [19]. Epsilon-toxin causes the rapid necrosis of sensitive cells. We previously demonstrated that the oligomerization of epsilon-toxin is promoted by ceramide production in the plasma membrane via activation of neutral sphingomyelinase induced by the toxin [18]. Moreover, we have shown that the epsilon-toxin is internalized into Madin-Darby Canine Kidney (MDCK) cells by endocytosis and induces the formation of intracellular vacuoles derived from late endosomes and lysosomes [20]. Two potential candidates for the toxin receptor have so far been reported: the cell membrane O-glycoprotein hepatitis A virus cellular receptor 1 (HAVCR1) [21,22], and the tetraspan transmembrane proteolipid myelin and lymphocyte protein (MAL) [6]. Epsilon-toxin receptors expressed in lipid raft microdomains contribute to assemble toxins, allowing for oligomer formation [19,23]. It has been described that caveolin-1 and -2 in plasma membrane lipid microdomains enhance epsilon-toxin-caused cytopathicity by facilitating the oligomer formation of epsilon-toxin [24]. The crystal structure of epsilon-toxin has a three-domain architecture strikingly similar to that of aerolysin [25]. Recently, the cryo-electron microscopy of epsilon-toxin pores revealed that the toxin assembles into a heptameric pore [26]. The toxin pore is 120 Å wide and 98 Å in height, with an inner diameter of 24 Å [26]. 

Calcium ions (Ca^2+^) are extensively involved in many cellular processes, including cytoskeleton reorganization, vesicular transport, gene expression regulation, and apoptosis [27,28,29]. Changes in intracellular calcium level pave the way for the modulation of cellular functions [30,31]. For instance, *Staphylococcus aureus* alpha-toxin has been reported to cause an increase in intracellular calcium in various cells [32,33]. *C. perfringens* enterotoxin and *Aeromonas sobria* hemolysin induced a rise in intracellular calcium, which resulted in the rapid development of cytopathic effects [34,35]. Phospholipase C (PLC), an important regulatory enzyme, catalyzes the hydrolysis of phosphatidylinositol-4,5-bisphosphate into inositol 1,4,5-triphosphate (IP_3_) and diacylglycerol (DAG) in response to various stimuli. A PLC-dependent pathway has been implicated in the assembly of the tight junction (TJ) [36]. IP_3_ then causes calcium release from the endoplasmic reticulum, resulting in an increase in intracellular calcium [37]. It has been previously described that epsilon-toxin causes a rise in intracellular Ca^2+^ concentrations in Madin-Darby canine kidney (MDCK) cells and renal mpkCCDc14 collecting duct cells [19,38]. However, how the epsilon-toxin-induced elevation of intracellular Ca^2+^ concentrations modifies the cytotoxic effects has not yet been defined. 

The purpose of the current study was to elucidate the MDCK cell monolayer response after treatment with epsilon-toxin. We examined the effect of epsilon-toxin on the membrane integrity of the MDCK cells. MDCK cells are highly sensitive to epsilon-toxin and are frequently used to assess the membrane permeability of various agents. We found that the calcium influx from extracellular medium or its release from intracellular stores induced by epsilon-toxin triggers changes in cellular function.

## 2. Results

### 2.1. Epsilon-Toxin Induces Barrier Dysfunction in MDCK Cells

**Figure 1:**
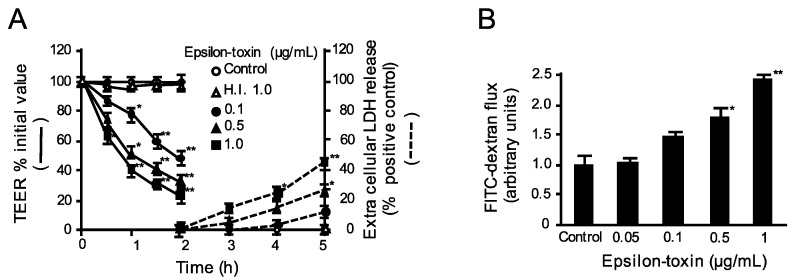
Epsilon-toxin-induced barrier dysfunction in Madin-Darby Canine Kidney (MDCK) cells. (**A**) Effects of epsilon-toxin on transepithelial electrical resistance (TEER). MDCK cells were apically inoculated with epsilon-toxin or heat-inactivated (H.I.) epsilon-toxin for various time periods at 37 °C. Lactate dehydrogenase (LDH) activity and TEER were examined. The results are described as the percentage of the baseline value. Data are expressed as means ± standard deviation (SD) (n = 4). * *p* < 0.05, ** *p* < 0.01. (**B**) Epsilon-toxin caused an increase in permeability to fluorescein isothiocyanate (FITC)-dextran. MDCK cells were incubated with varying concentrations of epsilon-toxin for 1 h. FITC-dextran permeability was identified. The values represent the mean ± standard deviation (SD) of four experiments. * *p* < 0.05, ** *p* < 0.01.

We investigated the effects of epsilon-toxin on barrier function in MDCK cell monolayers. Treatment of the apical surface of MDCK cell monolayers with epsilon-toxin caused a concentration-dependent decrease in transepithelial electrical resistance (TEER) (Figure 1A). The application of MDCK cells to epsilon-toxin (1000 ng/mL) led to a decline in the early TEER value by approximately 25% after 2 h of exposure. No further TEER decease by the toxin (1000 ng/mL) was recognized after 2 h. Lactate dehydrogenase (LDH) activity in the media was used as an index of cell death (Figure 1A). The exposure of MDCK cells to epsilon-toxin concentration dependently increased LDH beyond 3 h, indicating that the reduction in TEER induced by epsilon-toxin was not caused by cell death. In turn, epsilon-toxin was evaluated for paracellular marker permeability of MDCK cell monolayers by using fluorescein isothiocyanate-dextran20 (FD-20) as an indicator of large molecules. Epsilon-toxin accelerated the FD-20 permeability in MDCK cells in a dose-dependent manner (Figure 1B). These results indicate that epsilon-toxin damaged the barrier function of MDCK cells before cell death.

### 2.2. Epsilon-Toxin Activates Phospholipase Cγ-1 in MDCK Cells

**Figure 2:**
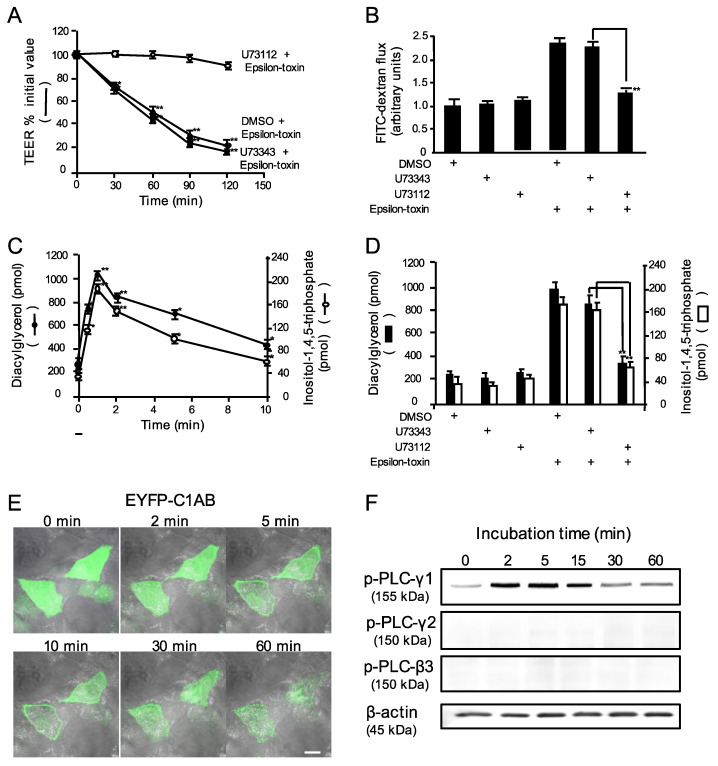
Effect of epsilon-toxin on phospholipase C. (**A**,**B**) MDCK cell monolayers on Transwell membranes were preincubated with vehicle control (dimethyl sulfoxide, DMSO), 10 μM U73122, or 10 μM U73343 for 1 h at 37 °C, and treated with epsilon-toxin (1 μg/mL) for various time periods at 37 °C. (**A**) TEER were examined. The results are described as the percentage of the baseline value. The values represent the mean ± standard deviation (SD) of four experiments. * *p* < 0.05, ** *p* < 0.01. (**B**) FITC-dextran permeability was examined. The values represent the mean ± standard deviation (SD) of four experiments. ** *p* < 0.01. One-way analysis of variance was employed to assess significance (**D**). (**C**,**D**) The effect of epsilon-toxin on the formation of diacylglycerol and IP_3_ in MDCK cells. (**C**) Cells were treated with epsilon-toxin (1 μg/mL) for the indicated time periods at 37 °C. (**D**) MDCK cells were preincubated with vehicle control (DMSO), 10 μM U73122, or 10 μM U73343 for 1 h at 37 °C, and treated with epsilon-toxin (1 μg/mL) for 1 min at 37 °C. Diacylglycerol (DAG) and IP_3_ contents were measured. The values represent the mean ± standard deviation (SD) of four experiments. * *p* < 0.05, ** *p* < 0.01. One-way analysis of variance was employed to assess significance (**D**). (**E**) The production of DAG on MDCK cell membrane treated with epsilon-toxin. Cells were transfected with EYFP-C1AB plasmid. A total of 24 h after transfection, cells were treated with epsilon-toxin (1 μg/mL) for various time periods at 37 °C. Cells were imaged using confocal fluorescence microscopy. Scale bar: 10 μm. (**F**) The phosphorylation of PLC in MDCK cells treated with epsilon-toxin. MDCK cells were treated with epsilon-toxin (1 μg/mL) for various time periods at 37 °C. Cell lysates were detected at the indicated times by immunoblotting with specific antibodies. Representative examples from one of four experimental groups were indicated.

The PLC-coupled signaling cascade plays a crucial role in the maintenance of TJ integrity [36]. To determine whether the epsilon-toxin-induced alterations of junctional integrity are due to the activation of PLC, we evaluated the effect of a PLC inhibitor (U73122) on the disruption to barrier function caused by epsilon-toxin. MDCK cells were preincubated with 5 μM U73122, followed by treatment with epsilon-toxin. The epsilon-toxin-caused TEER decrease and increase in FD-20 permeability were attenuated by pre-treatment with U73122 but were not affected by treatment with its inactive analogue, U73343 (Figure 2A,B). Then, to examine whether epsilon-toxin activates endogenous PLC, we determined the epsilon-toxin-induced diacylglycerol (DAG) and inositol 1,4,5-triphosphate (IP_3_) production in MDCK cells. Figure 2C shows that the toxin-induced formation of DAG and IP_3_ in a time-dependent manner. The formation of DAG and IP_3_ started within 30 sec of commencing treatment and peaked at 1 min after stimulation. Next, we examined whether the prior incubation of MDCK cells with U73122 influences both epsilon-toxin-induced DAG and IP_3_ formation (Figure 2D). The epsilon-toxin-induced formation of DAG and IP_3_ responses in MDCK cells pretreated with U73122 resulted in the inhibition of the responses caused by epsilon-toxin. In contrast, U73343 did not block the toxin-induced responses. To determine DAG formation in the cytoplasmic membrane of MDCK cells, we utilized an indicator, enhanced yellow fluorescent protein (EYFP)-tagged C1A and C1B regions of protein kinase C-γ (EYFP-C1AB), which permits the binding of DAG [39]. The transfection of MDCK cells with EYFP-C1AB resulted in homogeneous fluorescence signals throughout the cells (Figure 2E). Application of epsilon-toxin promoted the migration of EYFP-C1AB from the cytoplasm to the cell membrane in a time-dependent manner, indicating that the epsilon-toxin leads to DAG production in MDCK cells. Next, we determined the phosphorylation state of PLC isoenzymes after treatment with epsilon-toxin. Cells were incubated with epsilon-toxin during different time periods at 37 °C (Figure 2F). Phosphorylation of PLC-γ1 showed a maximum response at 5 min in the experimental conditions. In contrast, PLC-γ2 and PLC-β3 were not phosphorylated by epsilon-toxin. Therefore, it appears that epsilon-toxin induced DAG and IP_3_ production via PLC-γ1 activation.

### 2.3. Epsilon-Toxin Induces Ca^2+^ Influx in MDCK Cells

**Figure 3:**
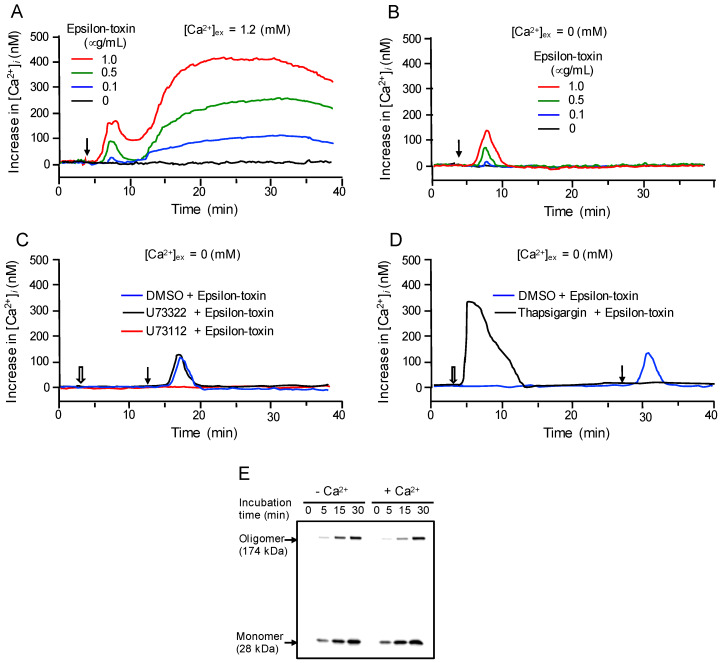
Effect of epsilon-toxin on intracellular calcium ([Ca^2+^]_i_) in MDCK cells. (**A**) MDCK cells were loaded with fura 2 acetoxymethyl ester (Fura 2 AM). [Ca^2+^]_i_ was measured. Elevation in [Ca^2+^]_i_ caused by epsilon-toxin was determined in cells with 1.2 mM Ca^2+^ in the extracellular medium. Epsilon-toxin was added at the arrow. (**B**) is similar to (**A**), except that the experiments were carried out in Ca^2+^-free medium. (**C**) In Ca^2+^-free medium, 10 μM U73112, 10 μM U73322, or DMSO were added at the white arrow, followed by epsilon-toxin (1 μg/mL) at 10 min. (**D**) Similar to (**C**), except that 10 μM thapsigargin or DMSO were added at the white arrow, followed by epsilon-toxin (1 μg/mL) at 20 min. The data show a typical result from four independent experiments. (**E**) MDCK cells were treated in either 1.2 mM Ca^2+^ or Ca^2+^ -free solution with epsilon-toxin (5 μg/mL) for the indicated times. After washing with phosphate-buffered saline (PBS), cells were lysed, and Ib and Ib oligomer were detected by Western Blotting using anti-Ib antibody. The data show a typical result from four independent experiments.

IP_3_, one of the second messengers generated by PLC-γ1 activation, is known to increase intracellular calcium [40,41]. Because the elevation of intracellular calcium (Ca^2+^) concentrations affects the barrier integrity in TJ [42,43], we investigated the epsilon-toxin induced-Ca^2+^ influx into MDCK cells. Epsilon-toxin evoked a rise in intracellular Ca^2+^ levels ([Ca^2+^]_i_) in a time- and concentration-dependent manner (Figure 3A). [Ca^2+^]_i_ rapidly increased, reaching a maximum within 2 min (early phase), and then decreased. After 10 min of incubation, [Ca^2+^]_i_ the increase began again and revealed a maximum response at approximately 20 min of incubation (late phase). In Ca^2+^-free solution, the toxin induced only the early phase response (Figure 3B), suggesting that epsilon-toxin stimulated intracellular Ca^2+^ release and Ca^2+^ influx from the extracellular space. To examine whether the Ca^2+^ release was correlated with the PLC signaling pathway, U73122 was utilized to block the pathway. After treatment with U73122, the epsilon-toxin-induced early phase calcium [Ca^2+^]_i_ increase was eliminated. In contrast, U73322 had no effect (Figure 3C). These results demonstrated that the early phase was correlated with the PLC-γ1 activation. The internal source from which epsilon-toxin-mobilized Ca^2+^ was investigated. As shown in Figure 3D, in Ca^2+^-free solution, thapsigargin, an endoplasmic reticulum Ca^2+^ pump inhibitor, led to a marked transient [Ca^2+^]_i_ increase followed by a decrease. Application of epsilon-toxin did not cause any [Ca^2+^]_i_ increase, indicating that the early-phase [Ca^2+^]_i_ increase induced by the toxin is derived from the endoplasmic reticulum. Then, we investigated the role of Ca^2+^ in the binding of epsilon-toxin to MDCK cells. When cells were incubated with epsilon-toxin in the presence of Hanks’ balanced salt solution (HBSS) buffer containing 1.8 mM Ca^2+^ for 15 and 30 min, 28 kDa (epsilon-toxin monomer) and 174 kDa (epsilon-toxin oligomer) bands were detected (Figure 3E). In contrast, the binding of the monomer and oligomer of the toxin to MDCK cells was observed in the Ca^2+^-free buffer, as well as in the buffer containing 1.8 mM Ca^2+^. These data indicate that epsilon-toxin binding and oligomer formation do not need extracellular calcium.

### 2.4. Epsilon-Toxin Changes the Organization of Junctional Proteins

**Figure 4:**
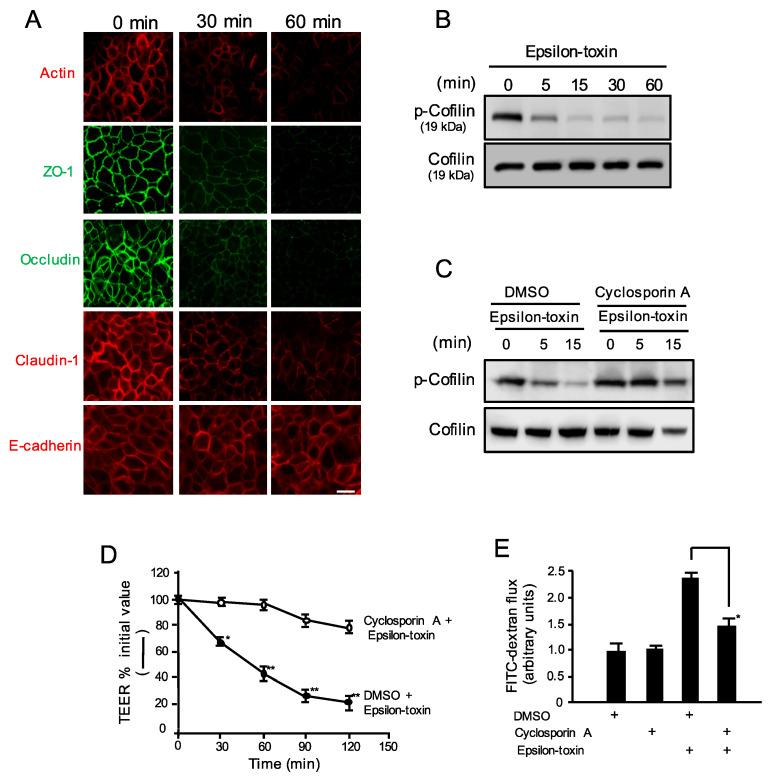
Effects of epsilon-toxin on MDCK cells. (**A**) The effects of epsilon-toxin on the distribution of actin, ZO-1, occludin, claudin-1, and E-cadherin in MDCK cells. MDCK cells were incubated with epsilon-toxin (1 μg/mL) for various time periods at 37 °C. Fluorescence antibody techniques were performed using antibodies against actin, occludin, ZO-1, claudin-1, and the N-terminal fragment of E-cadherin. Representative examples from one of four experimental groups were indicated. Bar: 10 μm. (**B**) Epsilon-toxin-induced cofilin dephosphorylation. MDCK cells were incubated with epsilon-toxin (1 μg/mL) for various time periods at 37 °C. (**C**) The effect of cyclosporin A on epsilon-toxin-induced cofilin dephosphorylation. MDCK cells were preincubated with vehicle control (DMSO), or 10 μM cyclosporin A for 1 h at 37 °C, and treated with epsilon-toxin (1 μg/mL) for various time periods at 37 °C. (**B**,**C**) Cellular lysates were detected by immunoblotting with antibodies. Representative examples from one of four experimental groups were indicated. (**D**,**E**) The effect of cyclosporin A on epsilon-toxin-induced barrier dysfunction in MDCK cells. MDCK cell monolayers were pretreated with vehicle control (DMSO) or 10 μM cyclosporin for 1 h at 37 °C and treated with epsilon-toxin (1 μg/mL) for various time periods at 37 °C. (**D**) TEERs were examined. The results are described as the percentage of the baseline value. The values represent the mean ± standard deviation (SD) of four experiments. * *p* < 0.05, ** *p* < 0.01. (**E**) The FITC-dextran permeability was identified. The values represent the mean ± standard deviation (SD) of four experiments. * *p* < 0.05. One-way analysis of variance was employed to assess significance (**D**).

As previously reported, Ca^2+^ entry induces TJ opening by pore-forming toxins [33,34,35]. We investigated the junctional protein expression levels, including claudin-1, ZO-1, and occludin (tight junction), E-cadherin (adherence junction), and actin of MDCK cells. Using immunofluorescence staining and confocal microscopy, we observed the localizations of junctional proteins after treatment with the toxin (Figure 4A). Exposure of MDCK cell monolayers to epsilon-toxin resulted in time-dependent declines in actin, ZO-1, occludin, and claudin-1, while the E-cadherin content was equal to control levels. These data indicate that epsilon-toxin induced decreases in actin, occludin, ZO-1, and claudin-1. However, epsilon-toxin did not cause a detectable decrease in E-cadherin.

### 2.5. Epsilon-Toxin Induces Cofilin Dephosphorylation

Ca^2+^ influx results in TJ opening, accompanied by cofilin activation and change in F-actin [44,45]. Cofilin activity is inhibited by phosphorylation at Ser-3 and is reactivated by dephosphorylation at Ser-3 [46]. We therefore examined whether epsilon-toxin treatment affects cofilin activity. MDCK cells were incubated with the toxin for various periods (Figure 4B). Cofilin dephosphorylation was observed at 5 min after the application of epsilon-toxin. The results show that epsilon-toxin activates cofilin in MDCK cells via dephosphorylation. It has been reported that Ca^2+^-induced cofilin dephosphorylation is mediated by the calcineurin (Cn)-dependent activation of protein phosphatase [46]. We therefore examined the effects of a Cn inhibitor, cyclosporine A (CsA), on the dephosphorylation of cofilin induced by epsilon-toxin. As shown in Figure 4C, CsA prevented the reduction in cofilin phosphorylation. Furthermore, the pretreatment of cell monolayers with CsA blocked the epsilon-toxin-induced decrease in TEER and the toxin-caused increase in permeability of FD-20. (Figure 4D,E).

## 3. Discussion

We found that epsilon-toxin (i) caused a barrier dysfunction of MDCK cells, (ii) stimulated calcium entry into the cells, (iii) raised the level of activated cofilin, and (iv) gave rise to the disruption of tight junction proteins. These discoveries that epsilon-toxin provokes epithelial barrier dysfunction through the entry of calcium provide new insights and may clarify the molecular mechanism of epsilon-toxin in pathogenicity. 

In *C. perfringens* type D-infected animals, epsilon-toxin causes severe kidney injury, the so-called “pulpy kidney disease” [2,19,47]. Epsilon-toxin accumulates predominantly in the mouse kidney [10,11], and the green fluorescent protein (GFP)-tagged toxin is bound to cryostat slices of kidneys of various species [12]. When epsilon-toxin was administered intravenously to mice, morphologic changes, but not severe renal damage, were detected in the cortex and the medulla of the kidneys [11]. Moreover, only a few kidney-derived cell lines were sensitive to the toxin (for example, MDCK cells, murine renal mpkCCDC14 cells and human renal G-402 cells) [48]. Renal damage appears to be a feature of the pathogenesis of the toxin. Although epsilon-toxin reduced TEER in confluent renal epithelial cells, as in MDCK or mpkCCDc14 cells, no changes in the tight junctions were observed between renal cells. This study revealed that epsilon-toxin actually influenced the barrier function of MDCK cells, as exhibited by obvious declines in the expression of a variety of junctional proteins, lowered TEER, and increase the permeability of FD-20 preceded by Ca^2+^ influx from extracellular medium.

It was previously shown that epsilon-toxin oligomerizes as a heptamer in target cells, and that the cytotoxic activity of the toxin is closely related to the oligomer formation [3,18,48,49,50]. Pore formation by bacterial pore-forming toxin in the host plasma membrane allows for the passage of low molecular weight molecules and ions. Entry of calcium ions through these pores affects diverse cellular processes, such as membrane repair with endocytosis [51], vesicle trafficking events [29], cytoskeletal reorganization, the modulation of gene expression, and apoptosis [27,28]. The alpha-toxin of *S. aureus* has been shown to cause the elevation of free intracellular calcium in several cell lines [32,33,52]. In cultured human intestinal Caco-2 cells, *Listeria monocytogenes* listeriolysin O [53], *C. perfringens* enterotoxin [34], *Aeromonas sobria* hemolysin [35] and *S. sureus* alpha-toxin [33] increased the intracellular calcium concentration, which may play a role in the cytotoxic effects [34,52]. The activation of endogenous PLC induces the hydrolysis of phosphatidylinositol 4,5-bisphosphate in the plasma membrane and the generation of diacylglycerol and inositol 1,4,5-trisphosphate (IP_3_) [54]. PLC activation also causes biphasic profiles of calcium mobilization [55]. In this study, epsilon-toxin simultaneously induced the production of DG and IP_3_ in MDCK cells, indicating that the toxin stimulated endogenous PLC. U73122, an endogenous PLC inhibitor, inhibited the epsilon-toxin-induced decrease in membrane permeability and the toxin-activated PLC activity. Moreover, epsilon-toxin elicited a biphasic increase in cytosolic [Ca^2+^]_i_, with an initial transient rise (early phase) followed by a sustained response (late phase). The early phase response, but not the late phase response, was unaffected in the Ca^2+^-free solution. The early phase of calcium mobilization was attributable to IP_3_, which evoked calcium release from intracellular stores. As the late phase of calcium entry was abolished in Ca^2+^-free solution, the intracellular concentration of calcium may be increased by the influx via toxin pores. Moreover, the toxin-induced Ca^2+^ response was inhibited by U73122. These results indicate that epsilon-toxin-induced PLC activation resulted in a biphasic process of calcium response. 

The elevation of intracellular Ca^2+^ decreases tight junction resistance [42]. Tight junction opening is associated with the dephosphorylation/activation of cofilin and reorganization of actin [44,45,46]. Cofilin is a potent regulator of actin filament dynamics [56]. Cofilin activity is inhibited by phosphorylation at Ser-3 by LIM kinase and is reactivated by dephosphorylation at Ser-3 by slingshot. The phosphorylation of cofilin results in the accumulation of actin fibers, while dephosphorylation results in their net loss. Calcineurin (Cn), a Ca^2+^/calmodulin (CaM)-dependent protein phosphatase, also called protein phosphatase 2B, was shown to mediate Ca^2+^-induced slingshot activation and cofilin dephosphorylation [46]. In this study, epsilon-toxin induced cofilin activation and actin alteration in MDCK cells. CyA, which inhibits Cn activity, blocked the toxin-induced decrease in TEER and dephosphorylation of cofilin. Moreover, when the cells were incubated with epsilon-toxin, the levels of ZO-1, occludin, and claudin-1 were decreased. Therefore, epsilon-toxin-induced Ca^2+^ influx, the activation of cofilin, and the subsequent decreases in actin, ZO-1, occludin, and claudin-1 were related to the increase in TJ permeability.

## 4. Conclusions

We revealed a novel mechanism by which epsilon-toxin decreases tight junction resistance. Epsilon-toxin causes the activation of PLCγ-1 in target cells. The activated PLCγ-1 leads to an increased production of IP_3_. This IP_3_-evoked intracellular Ca^2+^ increase activates cofilin, resulting in decreased actin, ZO-1, occludin and claudin-1. The present study also showed that Cn is involved in the TJ permeability increase by epsilon-toxin. Our data demonstrate that epsilon-toxin induces tight junction opening via a unique mechanism.

## 5. Materials and Methods

### 5.1. Materials

Epsilon-toxin purification was performed in accordance with a previous report [20]. An antiserum raised against epsilon-toxin was obtained using the previously described method [20]. U73112, U73343, thapsigargin, FITC-dextran 20 kDa, purified 1,2-diacylglycerol kinase from *Escherichia coli*, fluorescein isothiocyanate (FITC)-conjugated goat anti-mouse IgG, and bovine serum albumin (BSA) were purchased from Merck Japan (Tokyo, Japan). Antibodies against anti-phospho-PLC-γ1 (1248-Ser), anti-phospho-PLC-γ2 (759-Tyr) and anti-phospho-PLC-β3 (537-Ser), anti-phospho-cofilin (3-Ser) (77G2), anti-cofilin and anti-beta-actin were obtained from Cell Signaling. Rabbit polyclonal anti-human N-terminal fragment of E-cadherin antibody was obtained from Santa Cruz Biotec (Tokyo, Japan). Mouse primary antibodies against occludin and ZO-1, rabbit primary antibody against claudin-1, rhodamine-phalloidin, goat anti-rabbit Alexa Fluor 568 secondary antibody, a Fura-2 calcium-imaging calibration kit, and a bicinchoninic acid (BCA) kit for protein determination were purchased from ThermoFisher (Tokyo, Japan). Enhanced chemiluminescence kits and horseradish peroxidase-labeled anti-rabbit antibody were from GE Healthcare (Tokyo, Japan). Inositol 1,4,5-triphosphate (IP_3_) ELISA kit was from MyBioSource (San Diego, CA, USA). Fura-2/AM was from Dojindo Molecular Tech (Kumamoto, Japan). Protease inhibitor (PI) cocktail, Hanks’ balanced salt solution (HBSS), and Dulbecco’s modified Eagle’s medium (DMEM) were obtained from Nacalai Tesque (Kyoto, Japan).

### 5.2. Cell Culture

MDCK cells from Riken Cell Bank (Tsukuba, Japan) were cultured in DMEM supplemented with 10% fetal calf serum (FCS), 1% penicillin-streptomycin (Nacalai Tesque), and 2 mM glutamine (FCS-DMEM). Cells were cultivated in a humidified atmosphere containing 5% CO_2_ at 37 °C. 

### 5.3. Transepithelial Electrical Resistance (TEER) Measurements

MDCK cells were inoculated into collagen-coated membrane inserts (Transwell, pore size, 3 μm, catalog no. 3415; Corning) at a concentration of 5 × 10^5^ cells/mL. The culture medium was replaced every three days. The establishment of polarized monolayers of MDCK cells at three weeks was confirmed by cell morphological feature and measurements of TEER utilizing a Millicell-Electrical Resistance System (ERS)-2 (Merck-Millipore) [57]. TEER baseline values at three weeks after inoculation were in the range of 1600–2100 ohms/cm^2^. Epsilon-toxin was incubated with monolayer cultures at 37 °C. To evaluate the effects of inhibitors, various inhibitors or vehicles were preincubated to both the basolateral and apical sides of monolayers for 1 h, and then epsilon-toxin was applied to the apical side.

### 5.4. Permeability of MDCK Monolayer

The permeability of MDCK monolayer culture was determined by measuring the permeability of FITC-dextran 20 kDa (FD-20) from the apical to basolateral compartment [57]. MDCK cells were cultured on Transwell collagen-coated permeable support inserts for three weeks. To determine the trafficking from the apical to basolateral chamber, epsilon-toxin and FITC-dextran (1.5 mg/mL) were inoculated into the apical chamber. After 1 h of treatment at 37 °C, supernatants were removed from the basolateral compartment, and passaged fluorescence intensity (excitation/ emission wavelengths of 485/538 nm) was determined utilizing a fluorescence spectrophotometer (Infinite^®^ 200 PRO, Tecan, Kawasaki, Japan). FITC-dextran concentrations were determined by utilizing a standard curve gained by serial twofold dilutions of FITC-dextran.

### 5.5. Leakage of Lactate Dehydrogenase (LDH) Activity

LDH leakage from MDCK cells was assessed by elevated LDH levels with an LDH cytotoxicity assay (Nacalai Tesque), using the manufacturer’s protocols. A positive control for cytosolic lysates was prepared using 0.3% Triton X-100 (overall cell cytotoxicity) [57].

### 5.6. Determination of Diacylglycerol

MDCK cells (2.0 × 10^7^ cells) were treated with epsilon-toxin (1 μg/mL) at 37 °C for various times. Reactions were stopped by the addition of chloroform/methanol (1:2, v/v). Diacylglycerol (DG) was extracted as previously described [58,59]. In brief, extracted DG was converted to [^32^P]phosphatidic acid (PA) by DG kinase from *Escherichia coli* in the presence of [γ^32^P]ATP. After the separation of [^32^P]PA by thin-layer chromatography, the [^32^P] activity was determined using liquid scintillation counting. The content of DG metabolized to [^32^P]PA was calculated using the specific activity of [γ^32^P]ATP, as described previously [58,59].

### 5.7. Determination of Inositol 1,4,5-Trisphophate

MDCK cells (2.0 × 10^7^ cells) were treated with epsilon-toxin (1 μg/mL) for various times at 37 °C. The incubation was terminated by ice-cold 10% perchloric acid. The samples were held at 4 °C for 30 min and then centrifuged at 2500× *g* for 30 min at 4 °C. Each supernatant was adjusted to pH 7 using 10 N KOH solution. The amount of inositol 1,4,5-trisphophate (IP_3_) in the supernatants was determined with an IP_3_ ELISA kit (MyBioSource, San Diego, CA, USA), according to the manufacturer’s instructions [58].

### 5.8. Western Immunoblotting

MDCK cells (5.0 × 10^6^ cells) were treated in the absence or presence of epsilon-toxin (1 μg/mL) at 37 °C. The cells were lysed with 0.1 mL lysis buffer (2% wt/vol SDS, 10% glycerol, 0.1 mM Na_3_VO_4_, PI cocktail dissolved in 50 mM Tris-HCl buffer (pH 7.5)). The cell lysates were sonicated for 30 s and heated for 4 min at 95 °C. For the detection of Ib oligomer, cell lysates were incubated at 37 °C for 5 min. The preparations were separated by 10% SDS-polyacrylamide gel electrophoresis, electroblotted onto polyvinylidene difluoride (PVDF) membrane (Immobilon, Millipore, Tokyo, Japan), blocked with immunoblot blocking reagent (Sigma-Aldrich, Tokyo, Japan), probed with the appropriate first antibody and horseradish peroxidase-conjugated secondary antibody, and detected by an enhanced chemiluminescence kit using densitometry (LAS-4000, Fujifilm, Japan) [18].

### 5.9. Confocal Imaging of Diacylglycerol

Plasmid for the enhanced yellow fluorescent protein-fused C1AB domain of protein kinase C-γ (EYFP-C1AB) was constructed [39]. For DG staining on the outer leaflet of the plasma membrane, the plasmid was electroporated into MDCK cells utilizing a Neon Transfection System (ThermoFisher). After transfection, the cells were inoculated onto poly-L-lysine coating glass slides (MatTek, Ashland, MA, USA) and incubated at 37 °C for 24 h [39]. The cells were treated with epsilon-toxin for the indicated times at 37 °C and evaluated with a Nikon A1 confocal microscopy.

### 5.10. Immunofluorescence Technique

MDCK cells were plated on poly-L-lysine coating glass slides and incubated in a 5% CO_2_ incubator at 37 °C for 3 weeks in FCS-DMEM. The cells were incubated with epsilon-toxin (1 μg/mL) for the indicated time periods at 37 °C. MDCK cells were fixed with 2% paraformaldehyde in phosphate-buffered saline (PBS) at room temperature for 15 min, and then permeabilized in 0.2% Triton X-100 at room temperature for 15 min. Next, the cells were blocked with PBS containing 2% BSA (2% BSA-PBS), and then incubated overnight with first antibodies at room temperature. Rabbit antibody against the N-terminal fragment of E-cadherin, rabbit antibody against claudin-1, rabbit antibody against β-actin, mouse antibody against occludin, and mouse antibody against ZO-1 were stained using second antibodies (FITC-conjugated goat anti-mouse IgG and Alexa Fluor 568-conjugated goat anti-rabbit IgG) in 5% BSA-PBS at room temperature for 1 h. The slides were observed under a fluorescence microscopy (BIOREVO BZ-X) [57]. Fluorescent images were acquired utilizing a camera and processed using Photoshop (Adobe).

### 5.11. Determination of Intracellular Ca^2+^ Concentrations

Cytosolic Ca^2+^ concentrations were measured using the Ca^2+^ indicator Fura-2/AM by the previously described methods [60]. Cells were loaded with Fura-2/AM (4.5 μM) at 37 °C for 60 min in loading solution (HBSS containing 1% FCS, 2.5 mM probenecid, and 0.05% Pluronic F-127). After washing with HBSS, cells were incubated in assay solution (HBSS containing 1% BSA and 2.5 mM probenecid) at 37 °C for 20 min. Fura-2 fluorescence in response to the toxin was measured at 37 °C by the ratiometric method (excitation at 340 and 380 nm, emission at 500 nm) utilizing an inverted fluorescence microscopy (Nikon TMD-300, Tokyo, Japan) using Aqua-cosmos software U11158-02 program (Hamamatsu Photonics). The acquired imaging level was standardized using a Fura2 Ca^2+^-imaging calibration kit [60].

### 5.12. Statistical Analysis

One-way analysis of variance (ANOVA), followed by Bonferroni’s multiple-comparison post-test, was utilized to compare the mean values. Data are expressed as means ± standard deviation (SD) (n = 4). *p* values of 0.05 or less were considered significant.
